# On the Use of Rotary-Wing Aircraft to Sample Near-Surface Thermodynamic Fields: Results from Recent Field Campaigns

**DOI:** 10.3390/s19010010

**Published:** 2018-12-20

**Authors:** Temple R. Lee, Michael Buban, Edward Dumas, C. Bruce Baker

**Affiliations:** 1 Cooperative Institute for Mesoscale Meteorological Studies, Norman, OK 73072, USA; michael.buban@noaa.gov; 2 NOAA ARL Atmospheric Turbulence and Diffusion Division, Oak Ridge, TN 37830, USA; ed.dumas@noaa.gov (E.D.); bruce.baker@noaa.gov (C.B.B.); 3 Oak Ridge Associated Universities, Oak Ridge, TN 37830, USA

**Keywords:** sUAS, atmospheric boundary layer, sensors

## Abstract

Rotary-wing small unmanned aircraft systems (sUAS) are increasingly being used for sampling thermodynamic and chemical properties of the Earth’s atmospheric boundary layer (ABL) because of their ability to measure at high spatial and temporal resolutions. Therefore, they have the potential to be used for long-term quasi-continuous monitoring of the ABL, which is critical for improving ABL parameterizations and improving numerical weather prediction (NWP) models through data assimilation. Before rotary-wing aircraft can be used for these purposes, however, their performance and the sensors used therein must be adequately characterized. In the present study, we describe recent calibration and validation procedures for thermodynamic sensors used on two rotary-wing aircraft: A DJI S-1000 and MD4-1000. These evaluations indicated a high level of confidence in the on-board measurements. We then used these measurements to characterize the spatiotemporal variability of near-surface (up to 300-m AGL) temperature and moisture fields as a component of two recent field campaigns: The Verification of the Origins of Rotation in Tornadoes Experiment in the Southeast U.S. (VORTEX-SE) in Alabama, and the Land Atmosphere Feedback Experiment (LAFE) in northern Oklahoma.

## 1. Introduction

Earth’s atmospheric boundary layer (ABL) has traditionally been difficult to sample, yet adequately representing the physical processes occurring within it is essential to weather forecasting. Surface-based observational platforms, e.g., weather stations and flux towers, only penetrate at most a few tens of meters into the ABL. Rawinsondes provide a snapshot of the ABL, as they are released only twice daily from locations that are unevenly distributed across the world [1,2,3]. Like rawinsondes, surface-based remote sensing instruments, including microwave radiometers (MWR) and Atmospheric Emitted Radiance Interferometers (AERI), for measuring atmospheric thermodynamic quantities, lidars, and sodars for deriving winds, provide profiles at only one point in space and have limited resolution near the ground [4,5]. Whereas radars provide better horizontal coverage than rawinsondes and remote sensing instruments, oftentimes radars overshoot the ABL and thereby do not sample it well. Thus, despite its proximity to the ground, the ABL still presents a significant observation gap. Closing this gap is essential in many weather forecasting applications. For example, knowledge of the lower atmospheric stability and strength of the elevated inversion atop the ABL is critical for severe weather development [6]. Additionally, knowing the depth of the freezing layer is critical for forecasting the type of winter precipitation [7,8]. Furthermore, improved sampling of the ABL is critical for improving ABL parameterizations, and the numerical weather prediction (NWP) models through data assimilation.

Therefore, in recent years, the use of small unmanned aircraft systems (sUAS) begun in order to sample the ABL, which would help fill in this significant observation gap [9,10,11,12,13,14,15]. sUAS have been increasingly used to measure thermodynamic quantities [16], wind speed and direction [10,15], aerosols [17,18], heat flux [19,20,21], etc. Traditionally, fixed-wing sUAS have been used in ABL research [22,23]. Only within about the past five years have rotary-wing sUAS been used for ABL research. Rotary-wing sUAS are advantageous because of, e.g., their ability to hover, ease of use, etc. [10]. These characteristics make them an ideal choice for quasi-continuous ABL monitoring. Before these rotary-wing sUAS can be reliably used, however, their performance and the sensors used therein must be adequately characterized. Once confidence in measurements from the sensors used on rotary-wing sUAS is achieved, the sensors can be used for reliable ABL measurements. 

In the present study, we focused on the use of rotary-wing sUAS to obtain information on spatial and temporal variations in near-surface thermodynamic fields. The aim of our study is two-fold. We first evaluated the accuracy and precision of thermodynamic sensors commonly used on rotary-wing sUAS, and evaluated the iMet-XQ sensor, which measures temperature, humidity, and pressure. We quantified the iMet-XQ sensors’ accuracy by comparing their measurements with known standards in our calibration chamber, and by comparing the iMet-XQs’ measurements with measurements obtained from more traditional ABL-observing platforms (i.e., tower measurements and rawinsondes). We evaluated the iMet-XQ sensors’ precision by comparing measurements from two iMet-XQ sensors flown on the same sUAS, and by comparing measurements from two iMet-XQ sensors flown on two different sUAS.

The second component of this work was to use the thermodynamic measurements from the iMet-XQ sensors to address the following research questions:

1. What additional information on the structure of the lower ABL can be obtained from the sUAS thermodynamic measurements?
2. Are finescale (i.e., <100 m) surface gradients in temperature and moisture detectable using sUAS and, if so, to what height are these gradients detectable?

We addressed the above research questions using sUAS measurements obtained during two recent field campaigns: The Verification of the Origins of Rotation in Tornadoes Experiment in the Southeast U.S. (VORTEX-SE) in northern Alabama, and the Land Atmosphere Feedback Experiment (LAFE) in northern Oklahoma. 

## 2. Materials and Methods

### 2.1. Platform and Sensor Description

We evaluated the performance of two rotary-wing sUAS: a DJI S-1000 and MD4-1000. Both aircraft were multi-rotor, having eight and four rotors, respectively (Table 1). The DJI S-1000 had a gross weight of 11 kg, whereas the MD4-1000 weighed 4 kg. Due to its lower mass, the MD4-1000 had a payload capacity of 1.2 kg, whereas the DJI S-1000 could carry up to 4.5 kg. The NOAA Air Resources Laboratory (ARL) Atmospheric Turbulence and Diffusion Division (ATDD) has a Memorandum of Understanding (MOU) with the Federal Aviation Administration that allows for ATDD to operate the DJI S-1000 and MD4-1000 aircrafts at altitudes up to 1200 feet above ground level in Class G airspace. In calm wind conditions, the DJI S-1000 can operate for approximately 15–20 min, whereas the MD4-1000 can operate for approximately 25 min. Flight endurance decreases as a function of wind speed for both aircraft. More details about the aircraft appear in [24].

**Figure 1:**
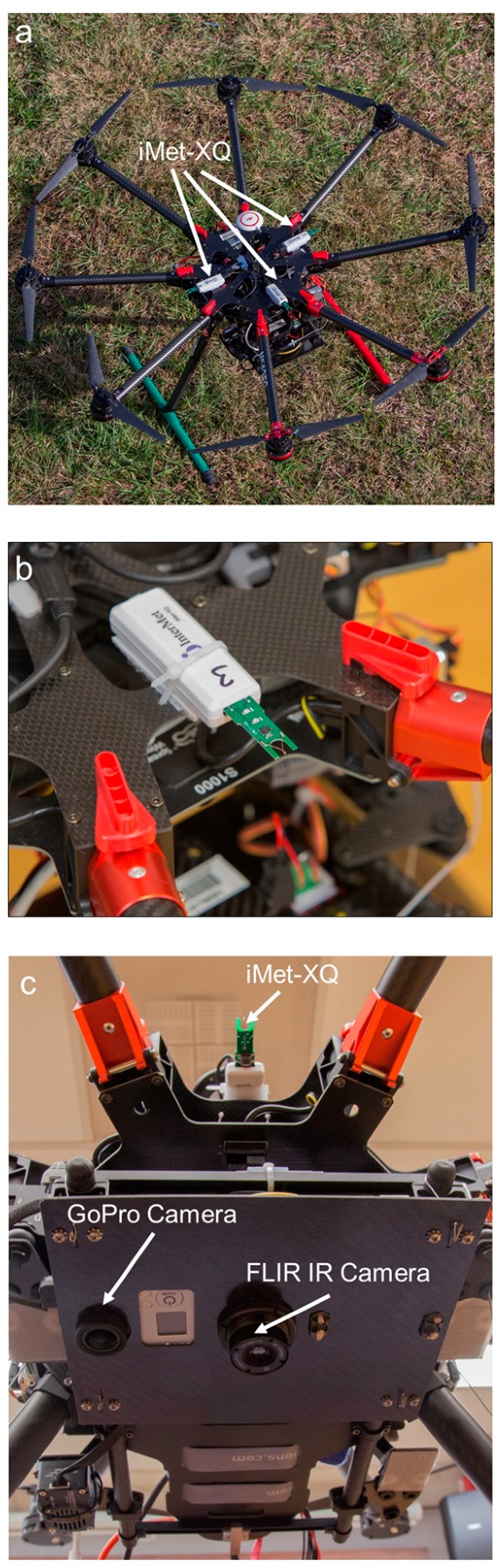
Sensor mounting locations of the iMet-XQ, FLIR infrared camera, and GoPro camera on the DJI S-1000. Panel (**a**) shows the position of three iMet-XQ sensors on board the sUAS prior to flight, panel (**b**) shows a closeup of one of the iMet-XQ sensors, and panel (**c**) shows the location of one iMet-XQ sensor relative to the GoPro and FLIR infrared cameras.

Each sUAS can carry two iMet-XQ sensors (International Met Systems Inc., Grand Rapids, MI, USA). The iMet-XQ sensors were used to measure temperature, relative humidity, and air pressure at manufacturer-stated accuracies of ±0.3 °C, ±5%, and ±1.5 mb, respectively, at 1-Hz frequency. During the sUAS flights, the iMet-XQ sensors were mounted 180° (90°) apart when two (three) iMet-XQ sensors were affixed onto the top of the sUAS platform (Figure 1a,b). Both sensors were unshielded but were aspirated by the propeller downwash to minimize radiation errors, as well as any potential heating effects caused by the thermal mass of the sUAS.

On the underside of the DJI S-1000, we used a FLIR Tau 2 infrared (IR) camera (FLIR Systems Inc., Wilsonville, OR, USA), which has a 7.5-mm lens, 90° × 69° view angle, and 336 × 256 pixel resolution [21,24,25] (Figure 1c). Surface temperature measurements from the IR camera were used, in conjunction with flux measurements derived from tower-mounted eddy covariance systems, to estimate the spatial variability in sensible heat flux following the technique developed in [21]. A GoPro Hero 3 camera with a 1024 × 768 pixel resolution was also installed onto the underside of the DJI S-1000 and used to sample land surface imagery at a 30-Hz frequency.

### 2.2. Calibration and Validation Techniques of Sensors and Platforms

Initial evaluations of the iMet-XQ sensors were performed using the NOAA/ARL/ATDD’s National Institutes for Standards and Technology (NIST)-traceable Thunder Scientific model 2500 two-pressure humidity generator. This chamber is a self-contained facility capable of producing known humidity values using the fundamental principle of the “two-pressure” generator developed by NIST (Thunder Scientific Operation and Maintenance Manual). Three temperature (10 °C, 20 °C, and 30 °C), and five relative humidity (20%, 40%, 60%, 80%, and 94%) set points were used for a total of 15 temperature/humidity combinations. The iMet-XQ sensors were installed in the chamber, and their response was measured for each temperature/relative humidity set point. 

Test flights with the sUAS were conducted at one of NOAA/ARLATDD’s nearby testbeds, i.e., Knox County Radio Control (KCRC) and House Mountain Radio Control (HMRC). KCRC (35.9483 N, 84.2331 W) is located approximately 30 km west of Knoxville, Tennessee, and HMRC (36.1260 N, 83.7945 W) is about 25 km to the northeast of Knoxville. Both sites were ideal testbeds because of their relative location to the ATDD and because, as of this writing, our cooperative agreement with the FAA allows operation of both the DJI S-1000 and the MD4-1000 sUAS up to 1200 feet (365 m) above ground level (AGL) at both KCRC and HMRC. Test flights at both sites were used to help evaluate how the placement of the iMet-XQ sensors on the sUAS airframe, as well as the platform orientation, affected the temperature and moisture measurements from the iMet-XQ sensors. Additional tests were conducted at HMRC, although in the present manuscript we focus only on results from KCRC. During the flight tests at KCRC, the iMet-XQ sensors were compared with thermodynamic measurements obtained from an instrumented tower. Measurements on the tripod included an aspirated Thermometrics platinum resistance thermometer (PRT), RM Young propeller anemometer, Vaisala HMP110 temperature and humidity probe, and a Vaisala PTB101B pressure sensor. These data were recorded onto a Campbell Scientific CR3000 data logger every 10 s.

Further evaluations of the sUAS were conducted as a component of VORTEX-SE. VORTEX-SE is a multi-year field experiment focused on studying the characteristics associated with the genesis of severe weather that are unique to the Southeast US [26,27]. During the 2017 VORTEX-SE campaign, we performed 20 sUAS vertical profiles with the DJI S-1000 and MD4-1000 sUAS to further evaluate our sensors and to characterize the evolution of the stability of the near-surface layer. These flights, which are summarized in Table 2, were performed at the Cullman, Alabama supersite (34.1939 N, 86.7967 W) up to 700-feet AGL (213-m AGL), which was the highest height to which we were allowed to fly in this area per our cooperative agreement with the FAA. At the Cullman supersite and located approximately 300-m west of where the sUAS profiles were performed, was a 10-m tower outfitted with an array of meteorological instruments, summarized in Table 3, as well as an eddy covariance system to quantify fluxes of momentum, sensible heat, latent heat, and carbon dioxide. The measurements from the Vaisala HMP110 humidity and temperature probe, and aspirated PRTs installed 10-m AGL, were used to help evaluate the accuracy of the sensors on board the sUAS. In addition to comparing the sUAS thermodynamic sensors with the tower measurements, we also compared them with measurements from rawinsondes launched at Cullman during VORTEX-SE.

Although the DJI S-1000 was primarily used for flights during VORTEX-SE, on 28 April 2017 we performed four simultaneous flights with this aircraft and the MD4-1000. During these flights, the two aircraft were flown with a horizontal separation of approximately 50 m to further evaluate the sensors’ precision and to help address the representativeness of thermodynamic measurements obtained from on-board sensors during a single flight.

### 2.3. Land Atmosphere Feedback Experiment

The measurements from our sUAS also played a critical role in the Land Atmosphere Feedback Experiment (LAFE) [28]. LAFE was a field campaign to investigate interactions occurring between different land surface types and the overlying atmosphere, with the aim to improve turbulence parameterizations in numerical weather prediction models. Investigators from about a dozen universities and government organizations deployed an array of boundary layer profilers, including water vapor differential absorption lidars, Doppler lidar, and a temperature and water vapor Raman lidar, as well as micrometeorological towers, during the month-long campaign at the Department of Energy Atmosphere Radiation Measurement site (36.6079 N, 97.4871 W), located near Lamont, Oklahoma, in August 2017. ATDD installed three 10-m micrometeorological towers outfitted with an array of meteorological and eddy covariance instruments summarized in Table 2 over different land surface types along a southwest to northeast transect of about 2 km. Tower 1 was deployed in an early growth soybean crop; Tower 2 was installed in a native grassland; and Tower 3 was in a mature soybean field. A fourth micrometeorological tower, 4 m in height, was installed by colleagues from the University of Hohenheim over a mixed shrubland, and included a similar array of meteorological and eddy covariance instruments, although eddy covariance measurements were made at only one height.

**Figure 2:**
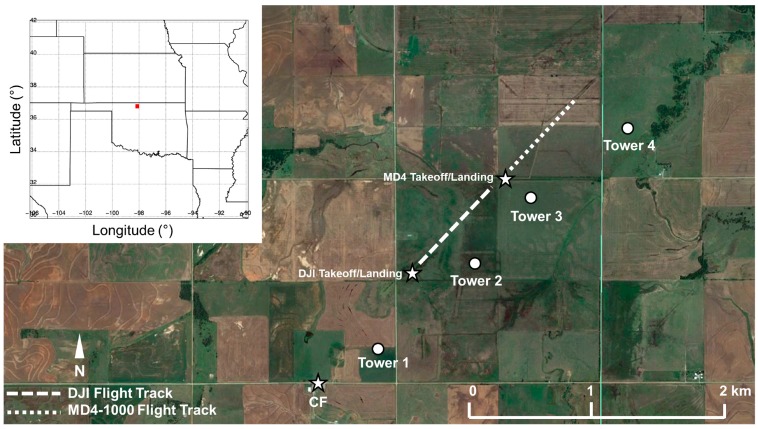
Relative location of flights of the DJI S-1000 (white dashed line) and MD4-1000 (white dotted line) sUAS to the four micrometeorological towers and central facility (CF) during LAFE. Image is courtesy of Google Earth. Inset map at the top left shows location of study site (red box) in north central Oklahoma.

During LAFE, we conducted 52 flights with the DJI S-1000 and MD4-1000 sUAS during three intensive observation periods (IOPs) on 14, 15, and 17 August 2017 (Table 2). We used our iMet-XQ sensors to measure temperature and relative humidity. The IR camera on the DJI S-1000 sUAS was used to characterize changes in land surface temperature and to estimate heat flux along the path of the sUAS. To this end, we performed sUAS flights in a box-like pattern along a southwest to northeast transect approximately 500 m west of Towers 2 and 3 (Figure 2). In these box-like patterns, the sUAS first ascended to 100-m AGL at a constant ascent rate of 1.5 m s^−1^, then flew horizontally at this same altitude for approximately 600 m from the takeoff location at a constant speed of 6 m s^−1^, at which point the sUAS ascended to 300-m AGL at 1.5 m s^−1^. The sUAS then returned to its takeoff location at this same altitude, again at 6 m s^−1^, before descending to the takeoff location at 1.5 m s^−1^. During ten of these flights, the DJI S-1000 and MD4-1000 were operated simultaneously, and while maintaining a horizontal separation between them of about 500 m. Due to the longer endurance of the MD4-1000 (discussed in Section 2.1), it was able to cover a longer distance of approximately 700 m, giving a total horizontal transect of up to nearly 1300 m using both aircraft. 

## 3. Sensor and Platform Evaluation

### 3.1. Sensor Accuracy

To have confidence in the accuracy of the iMet-XQ sensors, we compared their measurements with measurements made inside the ATDD’s calibration chamber, which we described previously in Section 2.2. We tested three iMet-XQ sensors in the chamber, and we aspirated the chamber using a Papst 12 V, 2.4 W fan. We found that the temperature measurements from all three iMet-XQ sensors were typically within ±0.1 °C of the NIST-traceable Thermometrics PRT standard. The three iMet-XQ sensors’ measurements of relative humidity were within ±5% of the NIST-traceable Vaisala humidity sensor. We also noted a cool bias in the iMet-XQ measurements of up to 0.3 °C at low temperature/relative humidity combinations, most notably at the 10 °C and 20% temperature/humidity set point. The largest differences between the iMet-XQ sensor humidity and NIST-traceable standards were found at high temperature/relative humidity combinations, i.e., for chamber temperatures >20 °C and chamber relative humidity >60%. Under these conditions, all iMet-XQ sensors had a dry bias, as the relative humidity was about 8% lower than the Vaisala standard.

We further evaluated the iMet-XQ sensors’ accuracy by comparing their measurements with in situ meteorological measurements from KCRC, as well as from Cullman as a component of VORTEX-SE. During the tests at KCRC, the iMet-XQ sensors were installed inside a shield adjacent to a Thermometrics platinum resistance thermometer and Vaisala HMT110 humidity probe and installed onto a micrometeorological tower at a height 2-m AGL. When all sensors were not aspirated using the fan installed inside the shield, R^2^ between the iMet-XQ measurements and the un-aspirated temperature and humidity measurements was 0.95 for temperature and 0.76 for humidity. During the tests in which all sensors were aspirated, which were representative of flight conditions, we found that R^2^ between the iMet-XQ measurements and the temperature, and relative humidity measurements was 0.94 for temperature and 0.99 for humidity. 

**Figure 3:**
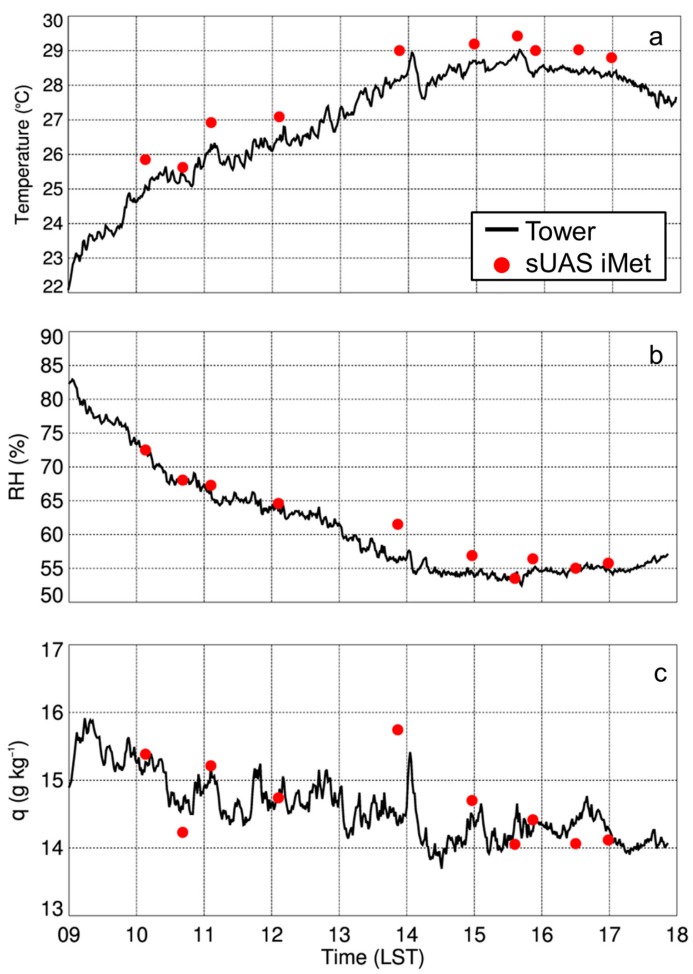
Temperature (**a**), relative humidity (**b**), and specific humidity (**c**) from the iMet-XQ sensors on the sUAS flown 28 April 2017 as a function of in situ measurements from the tower (black line) installed at Cullman, AL. sUAS measurements made represent the means of the lowest 5 m of the profile (red dots). Tower measurements were made at 2-m AGL and represent the 1-min means of 1-Hz samples.

Evaluations of the sensors during VORTEX-SE provided us with additional information on the performance of the iMet-XQ sensors. On 28 April 2017, we performed 10 flights at Cullman, AL, as a component of VORTEX-SE. During all flights, the sUAS ascended vertically at 1.5-m s^−1^ from the surface up to the maximum allowed altitude of 213-m AGL, per our cooperative agreement with the FAA. As discussed in Section 2.2 and briefly summarized here, these flights were conducted approximately 300-m east of the 10-m micrometeorological tower installed at Cullman. We then selected the measurements obtained during the ascending portion of the sUAS flight that corresponded with tower measurements from 2-m AGL, which was the height at which we had both a Vaisala HMP110 humidity and temperature probe and a PRT installed inside an aspirated shield (Table 3). During ten flights conducted with the DJI S-1000 and MD4-1000 sUAS, we found that the iMet-XQ temperature measurements were on average 0.65 °C ± 0.21 °C higher than the Cullman micrometeorological tower measurements (Figure 3a, Table 4). Relative humidity (Figure 3b) and specific humidity (Figure 3c) were within 5% and 1 g kg^−1^, respectively, of measurements obtained using the tower’s humidity probe, with the iMet-XQ showing mean differences that were 1.33 ± 1.57% and 0.10 ± 0.46 g kg^−1^ higher than the tower measurements. The warm bias in the measurements from the iMet-XQ sensor may be attributed to horizontal variations in temperature that can exist over the spatial scale between where the tower and sUAS measurements were made.

**Figure 4:**
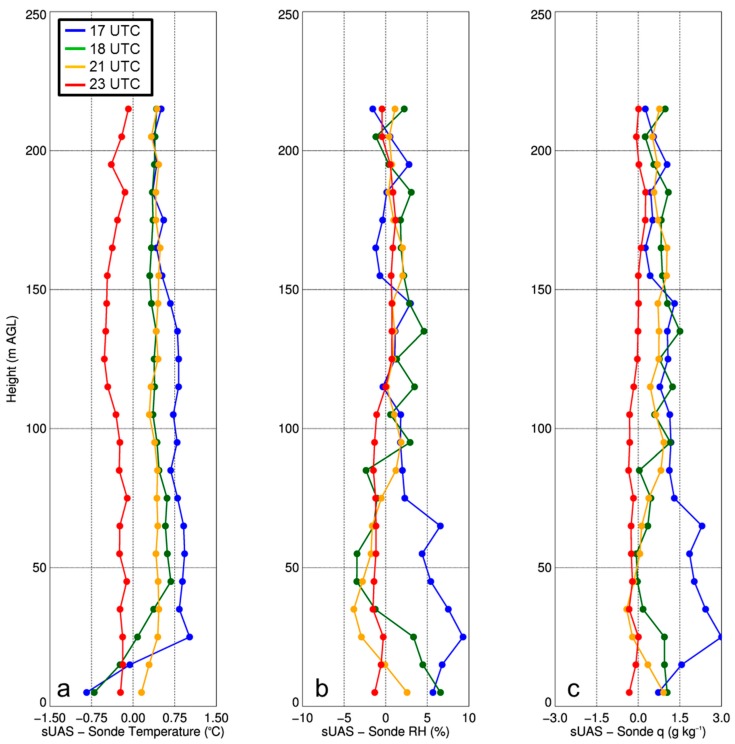
Difference between sUAS iMet-XQ temperature (**a**), relative humidity (RH) (**b**), and specific humidity (q) (**c**), and rawinsonde measurements at 1700 UTC, 1800 UTC, 2100 UTC, and 2300 UTC on 28 April 2017 from Cullman, AL. Data were averaged into 10-m bins.

Comparisons made between the iMet-XQ measurements obtained during the ascending portions of the sUAS flights and rawinsonde observations, also from 28 April 2017, yielded similar results. The mean differences between the iMet-XQ sensors installed on the sUAS and the rawinsonde observations were <0.6 °C for all flights (Table 4). Relative humidity generally agreed within ±2% between the iMet-XQ sensors and rawinsondes, but we did note that the flight at 1700 UTC had a wet bias of up to about 7% for relative humidity, which resulted in a bias in specific humidity of about 2.5 g kg^−1^ (Figure 4) In all cases, the differences were mostly random, rather than systematic, which was evident by the low R^2^ between the sUAS observations and rawinsonde observations, as shown in Table 4.

Thus, from the evaluations described in this section, we conclude that measurements from the iMet-XQ sensors agree with known standards within the manufacturer’s specifications, as indicated by the comparisons with measurements from inside the NIST-traceable chamber and through comparisons with platforms more traditionally used for making ABL measurements, i.e., those from fixed towers and rawinsondes. In the next section, we further evaluate the iMet-XQ sensors by quantifying their precision and determining optimal placement on rotary-wing sUAS.

### 3.2. Sensor Precision

**Figure 5:**
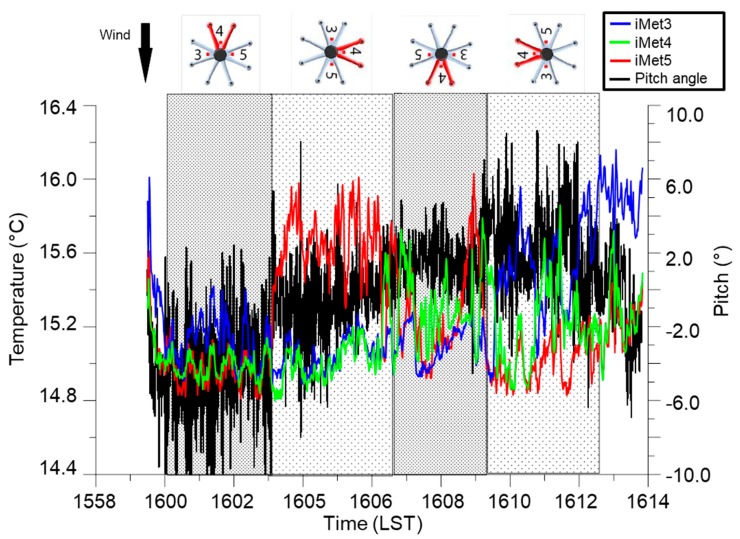
Inter-comparison among three iMet-XQ temperature sensors as a function of orientation into wind of the DJI S-1000 sUAS. Blue, green, and red lines show temperature measurements of the iMet-XQ sensors 3, 4, and 5, respectively, and the black line shows the pitch of the sUAS during flight. Positions of these sensors on the topside of the sUAS with respect to the sUAS orientation and wind direction are shown at the top of the figure.

To determine optimal sensor orientation during flight, we performed a series of test flights on 29 December 2015, in which the DJI S-1000 ascended to 20-m AGL adjacent to the KCRC tower. The DJI S-1000 sUAS hovered facing into the wind for 3 min, and was then rotated 90° every 3 min before descending. The three iMet-XQ sensors were installed onto the topside of the aircraft at the positions indicated at the top of Figure 5. When the sUAS was oriented into the wind and was pitching downward, there was good agreement among all three iMet-XQ sensors (Figure 5). However, when the sUAS was not oriented into the wind, we found larger differences in temperature among the three sensors. We attribute these larger differences in temperature to larger radiation errors induced by less aspiration of the sensors. 

**Figure 6:**
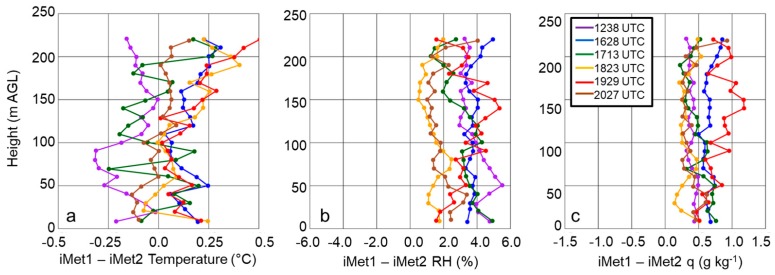
Difference between two iMet-XQ sensors; iMet-XQ 1 and iMet-XQ 2, in temperature (**a**), RH (**b**), and q (**c**) during six flights using a DJI S-1000 sUAS flown on 5 April 2017 at Cullman, AL. Purple, blue, green, orange, red, and brown correspond with flights made from 1238-1244 UTC, 1628-1639 UTC, 1713-1725 UTC, 1823-1834 UTC, 1929-1940 UTC, and 2027-2037 UTC, respectively. Data were averaged into 10-m bins.

Based on these tests, we concluded it was best to orient the sUAS into the wind during its flight. When this orientation was used, we found that this minimized the differences among the sensors’ measurements. For this reason, during all subsequent flights with the sUAS that we discuss in the present study, we installed the iMet-XQ sensors at positions 3 and 5 shown in Figure 5 when we flew the sUAS with two sensors on board, and we oriented the sUAS with a downward pitch into the wind. Operating the sUAS using this configuration resulted in differences between the iMet-XQ sensors that were within the manufacturer’s specifications, as shown in the example from 5 April 2017 at Cullman when we performed six flights with the DJI S-1000 sUAS. During these flights, we performed vertical ascents and descents at 1.5 m s^−1^ up to 213-m AGL, although only the data from the ascending portion of each flight were used following [21]. For all flights on this day, mean temperature differences were within ±0.2 °C (Figure 6a), mean relative humidity differences were within ±4% (Figure 6b), and mean specific humidity measurements for the profiles compared were within ±0.8 g kg^−1^ (Figure 6c, Table 4). The differences observed during these flights were comparable to differences found during other evaluations conducted (not shown).

**Figure 7:**
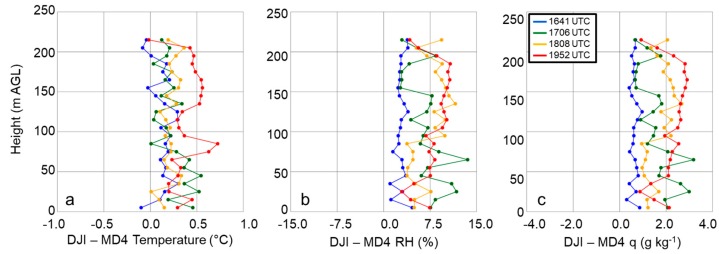
Comparison between measurements of temperature (**a**), relative humidity (**b**), and specific humidity (**c**) from the iMet-XQ sensors on the DJI S-1000, and those on the MD4-1000 sUAS. Both aircraft were flown simultaneously ≈50 m apart at Cullman 28 April 2017. Blue, green, orange, and red lines coincide with flights made 1641-1650 UTC, 1706-1714 UTC, 1808-1820 UTC, and 1952-2007 UTC, respectively. Data were averaged into 10-m bins.

Additional evaluations of the precision of the iMet-XQ sensors came from comparing thermodynamic measurements from both iMet-XQ sensors installed onto the topside, as discussed in Section 2.1 and shown in Figure 1, of both the DJI S-1000 and MD4-1000 sUAS. During these tests, the DJI S-1000 and MD4-1000 performed vertical ascents and descents at 1.5 m s^−1^ and were flown approximately 50-m apart during the 28 April 2017 case study at Cullman, AL as a component of VORTEX-SE. Prior to comparing the measurements from each aircraft, we first computed the mean of the measurements from the two iMet-XQ sensors at each sampling height, and we used only the data obtained during the sUAS ascent. Mean differences in temperature for each of the profiles obtained from the DJI S-1000 and MD4-1000 on this day ranged from 0.12–0.39 °C (Figure 7a); for relative humidity (Figure 7b), and specific humidity (Figure 7c), the differences ranged from 2.8–7.9% and 0.63–2.18 g kg^−1^, respectively. Furthermore, these comparisons showed that these differences did not depend on height relative to ground level, nor were there biases as a function of time of day. Because of the absence of any systematic bias here, these comparisons provided us with further confidence in the precision of the measurements obtained from the thermodynamic sensors installed on board our rotary-wing sUAS. 

**Figure 8:**
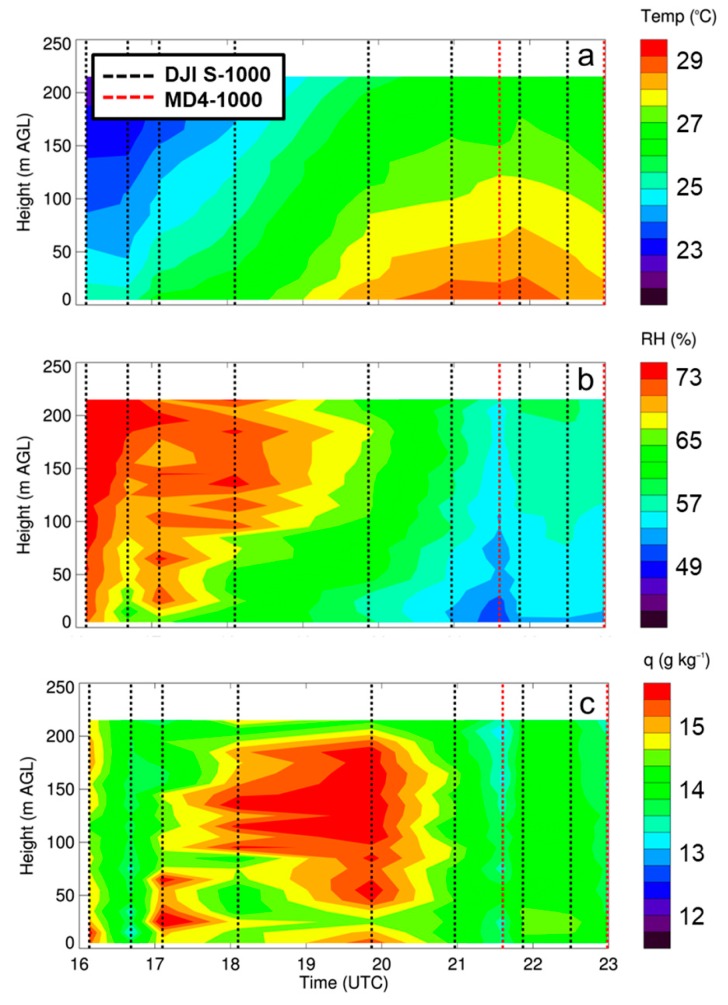
Evolution of temperature (**a**), relative humidity (**b**), and specific humidity (**c**) at Cullman, AL on 28 April 2017 obtained from the DJI S-1000 and MD4-1000 sUAS. Black and red dashed vertical lines represent profiles made by the DJI S-1000 and MD4-1000 sUAS, respectively.

To illustrate the capabilities of multiple vertical profiles using rotary-wing sUAS, we performed ten vertical profiles on 28 April 2017 with our sUAS, starting at 1000 local time (local time = UTC–6) and continuing through 1700 local time (Figure 8). For all flights, we used only the ascending portion of each profile, and used an ascent rate of 1.5 m s^−1^. This sequence of flights provided finescale details on the temperature and moisture structure of the lower ABL. With these measurements, we were able to observe, for example, the development of a near-surface super-adiabatic layer, and we observed a general increase in low-level moisture content, as evident by the increase in specific humidity between 1600 and 2000 UTC. Whereas this type of detailed information can be obtained from other observing platforms, sUAS provided much more finescale detail on the evolution of near-surface thermodynamic fields. For example, as discussed in Section 1 and briefly summarized here, remote sensing instruments like radars, AERIs, and MWRs have difficulty resolving thermodynamic fields near the surface. Additionally, tethersondes have limited altitude range and can be difficult to deploy in windy conditions. By performing these sUAS flights throughout the day, we can gain this same detailed information to obtain a more comprehensive view of the evolution of the lower ABL and spatial variability therein during other field experiments as well, which we discuss in the next section.

## 4. Spatiotemporal Variability of Near-Surface Thermodynamic Fields During LAFE

**Figure 9:**
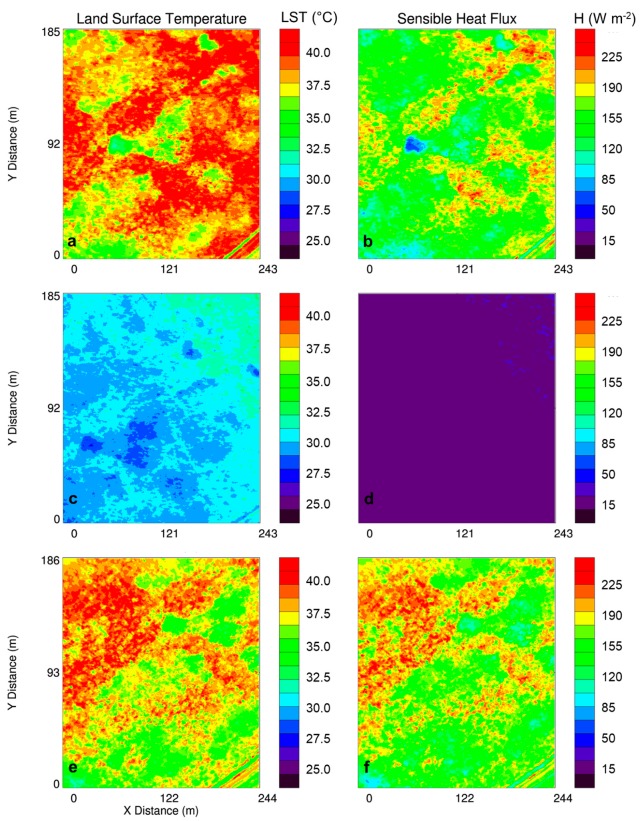
Land-surface temperature (LST) (**a**) and surface sensible heat flux (**b**) at 20:24 UTC on 14 August 2017, sampled from the downward-pointing IR camera on the DJI S-1000 sUAS while flying at a constant altitude of 300-m AGL. Same for panels (**c**) and (**d**), and for panels (**e**) and (**f**), but at 20:16 UTC 15 August 2017, and 20:16 UTC 17 August 2017, respectively. The same region is shown in all six panels.

**Figure 10:**
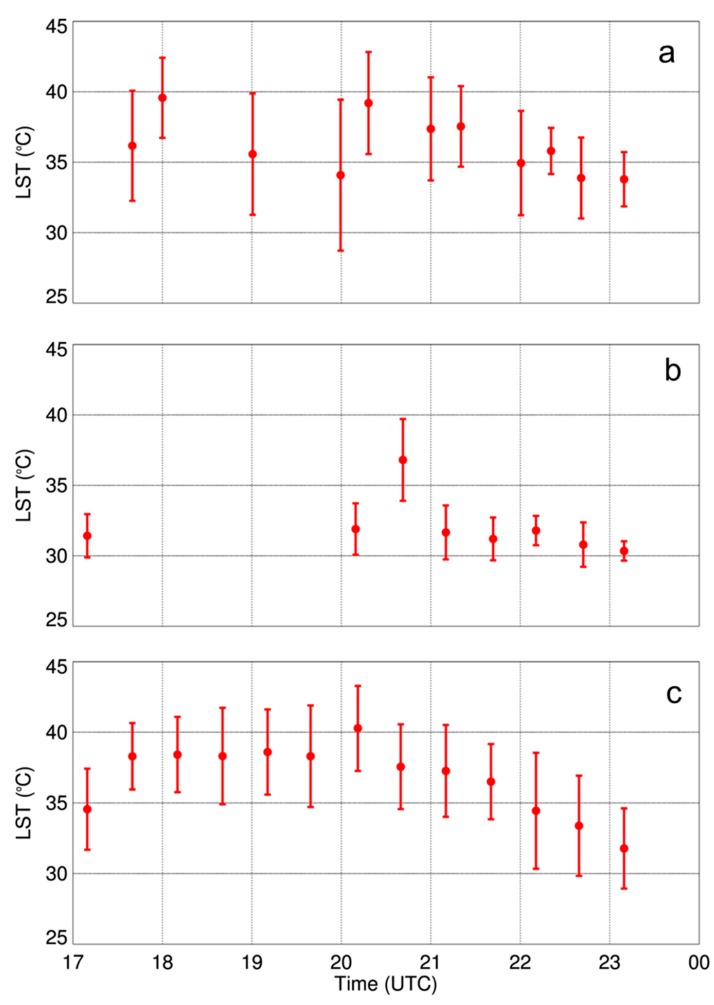
Mean (filled circles) ±1 standard deviation (bars) in LST obtained from the DJI S-1000 sUAS flights on 14 August 2017 (**a**), 15 August 2017 (**b**), and 17 August 2017 (**c**) during LAFE.

Multiple sUAS flights in a box-like pattern over the same area during LAFE, provided critical information on the spatiotemporal variability of temperature and moisture fields in the lower ABL. Synoptic analyses (not shown) for the period 14–17 August 2017, when over 50 sUAS flights were flown, indicated the presence of a stalled frontal boundary located over southeastern Oklahoma and a cold front to the northwest of the region on 14–15 August. Southeasterly winds between 3 and 5 m s^−1^ were observed at all three 10-m micrometeorological towers during this period. Synoptic analyses, corroborated by observations from the towers, showed that a cold front passed through the LAFE domain around 2330 UTC on 16 August, and was followed by northwesterly flow throughout much of the day on 17 August. As a result, of the three days on which we conducted sUAS flights during LAFE, 17 August was the clearest day. This observation was based on the day’s clearness index, which represents the ratio of the total amount of incoming shortwave radiation measured at a site and summed for a given day to the sum of the total maximum incoming solar radiation that could be received on that day at that particular location [29,30,31,32]. This was also corroborated by other LAFE participants at the site that observed clear skies. At Tower 2, the clearness index on 14, 15, and 17 August was 0.62, 0.32, and 0.73, respectively, which was consistent with the clearness indices calculated using observations from the other towers. As a result, we observed considerable differences in the surface temperature as well as temperature and moisture fields in the lower ABL. Within the footprint of the IR camera (i.e., 186 m × 244 m when 300-m AGL), land surface temperatures varied by up to 10 °C, with corresponding variations in surface sensible heat flux of up to 200 W m^−2^, during the middle afternoon on 14 and 17 August (Figure 9). In contrast, land surface temperatures over the field of view of the IR camera varied typically only by about 2–3 °C on 15 August. This variability was further evident by computing the standard deviation in land surface temperature for each flight on each day (Figure 10). Overall, the surface temperature variability was largest on 14 August, with standard deviations of 3–4 °C compared to about 1.5 °C the following day. On 17 August, there were notable consistencies in the flight-to-flight variability during the afternoon, following the frontal passage the previous night, with standard deviations of around 3 °C.

Thus, from these analyses, it becomes evident that there was considerable finescale variability in land surface temperature and heat flux. This variability can be attributed to finescale heterogeneities in the land surface, for example patches of bare ground surrounded by taller grass, and areas of more dense vegetation that was present using imagery obtained from the GoPro camera on board the DJI S-1000, site surveys, and Google Earth satellite imagery shown in Figure 2. To assess what role this finescale variability had on the lower ABL temperature and moisture fields, we used the iMet-XQ data from on board the DJI S-1000 and MD4-1000 sUAS. For each flight, we binned the data into 10 m bins and computed the means and standard deviations. Because of the box-like pattern in which the sUAS was flown, we used data from both the ascents and descents to capture the diurnal evolution of temperature and moisture up to 300-m AGL. Although we acknowledge there are differences in the iMet-XQ temperature and moisture measurements when comparing the ascending portions of the sUAS flights with the descending portion of the sUAS flights (not shown), we note that the differences we find are within the manufacturer’s stated level of accuracy for the iMet-XQ sensors.

**Figure 11:**
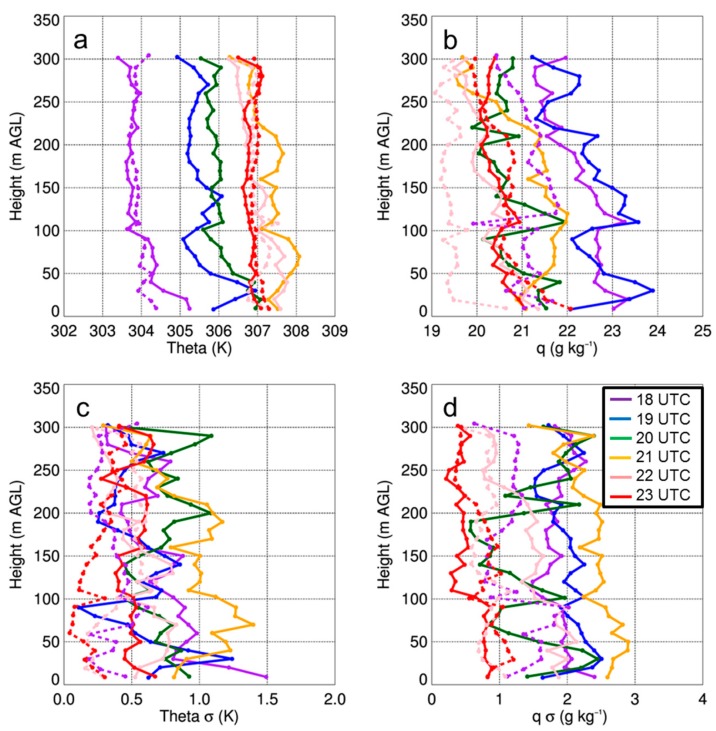
Vertical profiles of potential temperature (i.e., theta) (**a**) and q (**b**) as a function of height for select sUAS flights with the DJI S-1000 (solid) and MD4-1000 sUAS (dashed) on 14 August 2017. Panels (**c**) and (**d**) show the standard deviation (σ) in theta and q, respectively. Purple, blue, green, orange, pink, and red lines coincide with flights made starting at 1800 UTC, 1900 UTC, 2000 UTC, 2100 UTC, 2200 UTC, and 2310 UTC, respectively. Data were binned every 10-m AGL.

**Figure 12:**
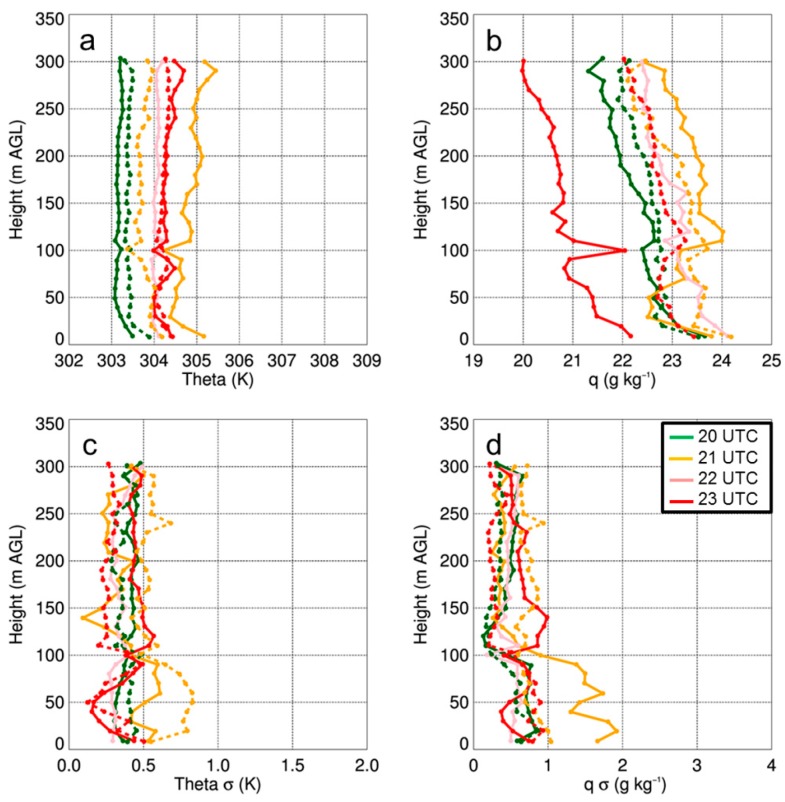
Vertical profiles of theta (**a**) and q (**b**) as a function of height for select sUAS flights with the DJI S-1000 (solid) and MD4-1000 (dashed) sUAS on 15 August 2017. Panels (**c**) and (**d**) show the standard deviation (σ) in potential temperature and specific humidity, respectively. Green, orange, pink, and red lines coincide with flights made starting at 2000 UTC, 2100 UTC, 2200 UTC, and 2310 UTC, respectively. Data were binned every 10-m AGL.

**Figure 13:**
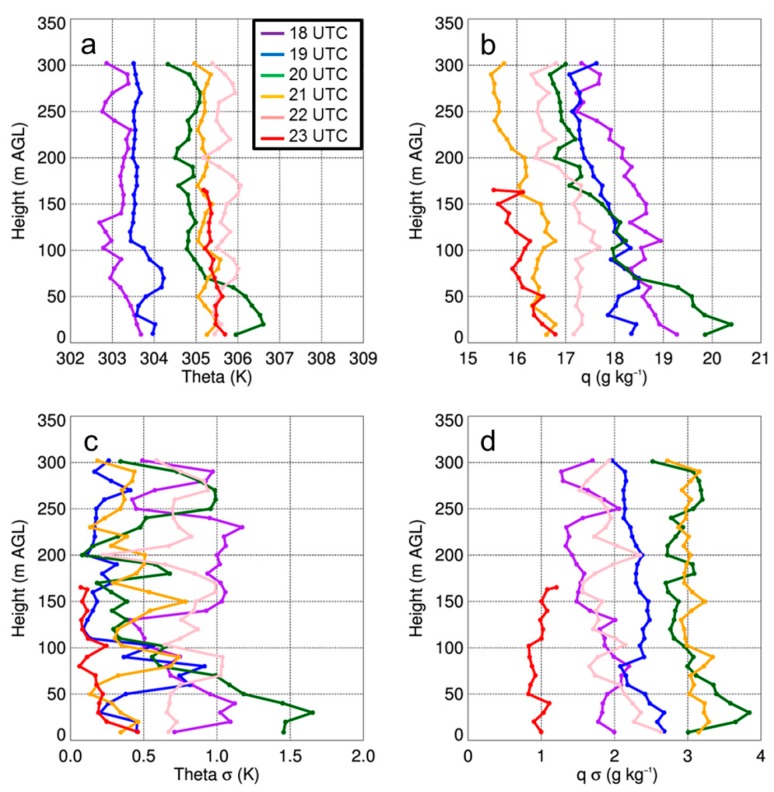
Vertical profiles of theta (**a**) and q (**b**) as a function of height for select sUAS flights with the DJI S-1000 on 17 August 2017. Panels (**c**) and (**d**) show the standard deviation (σ) in potential temperature and specific humidity, respectively. Purple, blue, green, orange, pink, and red lines coincide with flights made starting at 1810 UTC, 1910 UTC, 2010 UTC, 2110 UTC, 2210 UTC, and 2310 UTC, respectively. Data were binned every 10-m AGL.

Vertical profiles of temperature and moisture indicated a well-mixed ABL that occurred during the afternoon on all three days (Figure 11a, Figure 12a and Figure 13a), as evident by vertical gradients in potential temperature and specific humidity, which were generally <1 K and <2 g kg^−1^. The presence of small vertical gradients was not surprising given the time of day and meteorological conditions under which these profiles were made. Further evidence of the well-mixed daytime ABL was apparent when comparing the simultaneous vertical profiles from the DJI S-1000 and MD4-1000 sUAS that were flown ≈ 500-m apart. On the two days when there were simultaneous profiles (i.e., 14 and 15 August), potential temperature differences between the two sites were often <0.5 K up the maximum altitude to which the profiles were made. Water vapor showed larger variability than temperature, with differences between measurements from the DJI S-1000 and MD4-1000 of up to 2 g kg^−1^.

**Figure 14:**
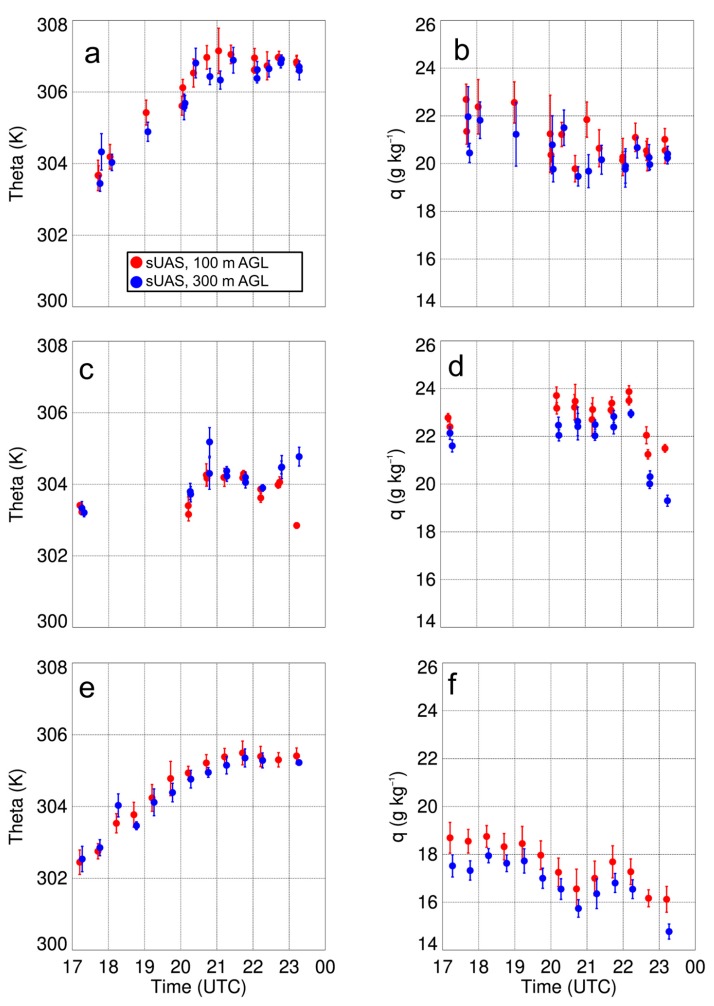
Mean and standard deviation of theta (**a**) and q (**b**) during the sUAS flights on 14 August 2017, while the sUAS was flown at 100-m AGL (red) and 300-m AGL (blue). Same for panels (**c**) and (**d**), and for panels (**e**) and (**f**), but for 15 and 17 August 2017, respectively.

From the vertical profiles of the standard deviations in the potential temperature and specific humidity fields, we found that there is typically more variability near the surface. Also, of the three days, the standard deviations were lowest on 15 August due to more cloud cover on this day. These profiles also allowed for us to determine if the variability was any different at 100-m AGL and 300-m AGL, where the sUAS was flown at a constant height for 500–700 m over the surface. However, we did not see any larger variability at these two specific heights than we observed at the other heights when the sUAS was moving vertically, and this was found to occur on all three days in the study period. Furthermore, we did not find large differences in the iMet-XQ measurements 100-m or 300-m AGL when the sUAS was flying over surfaces with different thermal characteristics (shown in Figure 9); the temperature and moisture variations remained within ±0.5 K and 0.5 g kg^−1^, respectively, during these horizontal transects. The presence of small temperature and moisture variations was further evident by computing the mean potential temperature from these flights as a function of time of day. Characteristic of a well-mixed daytime ABL, we observed small gradients in potential temperature at both 100-m and 300-m AGL (Figure 14). These findings indicated that the vertical variability in temperature, as well as in moisture, was comparable to the horizontal variability. In other words, the land surface variability was not detectable in either the temperature or moisture fields during the sUAS flights, as finescale surface thermal gradients were mixed out. This implies that, whereas finescale thermal gradients that can be up to ~10 °C over the scale of a few tens of meters, they are readily mixed out and are too small to have a detectable impact on lower level temperature or moisture fields.

## 5. Conclusions and Outlook

Small unmanned aircraft systems (sUAS) have promise to help close a critical observation gap in the ABL. In the present study, we implemented calibration and validation procedures to evaluate thermodynamic sensors used on two rotary-wing sUAS. Vertical profiles with the sUAS were then used during the LAFE and VORTEX-SE field experiments to provide finescale details on the evolution of the temperature and moisture structure of the lower ABL. More than 50 flights during LAFE, and 20 flights during the 2017 VORTEX-SE campaigns were used to provide additional details on the spatiotemporal evolution and variability of near-surface thermodynamic fields. Most notably, these flights, particularly those during LAFE, indicated that although finescale near-surface thermal gradients can be large, these gradients are oftentimes too small to have a detectable impact on low-level ABL temperature or moisture fields, as they are readily mixed out.

The results from this study provide the groundwork necessary to allow atmospheric data collected using flights from rotary-wing sUAS to become more reliable and routine. We envision a nationwide network of rotatory-wing sUAS providing near-continuous observations of not only temperature and moisture, but also wind speed and direction [10,15], within and above the ABL. This measurement suite will provide details of the ABL on a much finer scale than have previously been observed, which will help improve and refine existing ABL parameterizations currently used in NWP. Observations from sUAS have already begun to be assimilated into numerical models [33,34,35], and we envision that data from rotary-wing sUAS will ultimately be assimilated into NWP in real-time, thereby leading to improved weather forecasts. 

Another added benefit of routine sUAS profiles is that they may be used for air quality monitoring. Due to weight and power consumption requirements, instruments traditionally used to sample trace gas and aerosol species have not been well-suited for use on sUAS. However, as new sensors continue to be developed that are able to fit onto sUAS platforms, sUAS may also be used to provide critical information on trace gas and aerosol concentrations within the ABL [18]. Just as thermodynamic and kinematic measurements from sUAS may help improve NWP, trace gas and aerosol measurements from sUAS have the potential to improve air chemistry models and air quality forecasts. These measurements, in conjunction with the thermodynamic measurements discussed in the present study, will make rotary-wing sUAS a very powerful platform for ABL observation in the coming years.

## Figures and Tables

**Table 1:** sUAS characteristics.

Model	DJI S-1000	Microdrone MD4-1000
Registration	N542FC	N536JN
Manufacturer	DJI	Microdrone
Owner/Operator	NOAA/ARL/ATDD	NOAA/ARL/ATDD
Mission	Atmospheric profiling	Atmospheric profiling
Units in Fleet	1	1
Vehicle Type	Multi-rotor	Multi-rotor
Gross Weight	11 kg	3.85 kg
Wing Span	1.0 m	1.0 m
Length	1.0 m	1.0 m
Payload Capacity	4.5 kg	1.2 kg
Engine Type	8 electric motors	4 electric motors
Autopilot	DJI A2 with iOSD Mk II	Microdrone
Max Speed	10 m s^−1^	10 m s^−1^
Loiter Speed	0 m s^−1^	0 m s^−1^
Endurance	15 min	25 min
Ceiling	365 m	1000 m

**Table 2:** sUAS flights used in the present study. Note that, at both sites, local time = UTC–6.

Location	Aircraft	Date	Start Time (UTC)	End Time (UTC)	Flight Duration (s)
Cullman, AL	DJI S-1000	5 Apr 17	12:38:45	12:44:52	367
Cullman, AL	DJI S-1000	5 Apr 17	16:28:02	16:39:20	678
Cullman, AL	DJI S-1000	5 Apr 17	17:13:43	17:25:07	684
Cullman, AL	DJI S-1000	5 Apr 17	18:23:28	18:34:17	649
Cullman, AL	DJI S-1000	5 Apr 17	19:29:59	19:40:38	639
Cullman, AL	DJI S-1000	5 Apr 17	20:27:05	20:37:39	634
Cullman, AL	DJI S-1000	28 Apr 17	16:08:14	16:19:22	668
Cullman, AL	DJI S-1000	28 Apr 17	16:41:12	16:49:31	499
Cullman, AL	DJI S-1000	28 Apr 17	17:06:34	17:14:20	466
Cullman, AL	DJI S-1000	28 Apr 17	18:06:07	18:20:17	850
Cullman, AL	DJI S-1000	28 Apr 17	20:52:43	20:06:38	835
Cullman, AL	DJI S-1000	28 Apr 17	20:58:43	21:10:46	723
Cullman, AL	DJI S-1000	28 Apr 17	21:52:21	22:04:06	705
Cullman, AL	DJI S-1000	28 Apr 17	22:30:37	22:41:35	658
Cullman, AL	MD4-1000	28 Apr 17	16:41:28	16:50:28	540
Cullman, AL	MD4-1001	28 Apr 17	17:06:42	17:15:20	518
Cullman, AL	MD4-1002	28 Apr 17	18:06:15	18:21:06	891
Cullman, AL	MD4-1003	28 Apr 17	20:52:55	21:07:29	874
Cullman, AL	MD4-1004	28 Apr 17	21:36:10	21:45:59	589
Cullman, AL	MD4-1005	28 Apr 17	22:59:26	23:09:55	629
Lamont, OK	DJI S-1000	14 Aug 17	17:40:05	17:49:58	593
Lamont, OK	DJI S-1000	14 Aug 17	18:00:02	18:09:49	587
Lamont, OK	DJI S-1000	14 Aug 17	18:40:46	18:50:33	587
Lamont, OK	DJI S-1000	14 Aug 17	18:59:53	19:10:25	632
Lamont, OK	DJI S-1000	14 Aug 17	19:59:31	20:09:41	610
Lamont, OK	DJI S-1000	14 Aug 17	20:18:54	20:28:59	605
Lamont, OK	DJI S-1000	14 Aug 17	21:00:05	21:10:46	641
Lamont, OK	DJI S-1000	14 Aug 17	21:20:41	21:31:24	643
Lamont, OK	DJI S-1000	14 Aug 17	22:00:09	22:10:51	642
Lamont, OK	DJI S-1000	14 Aug 17	22:20:29	22:30:53	624
Lamont, OK	DJI S-1000	14 Aug 17	22:40:18	22:50:23	605
Lamont, OK	DJI S-1000	14 Aug 17	23:10:13	23:20:47	634
Lamont, OK	MD4-1000	14 Aug 17	17:01:55	17:07:39	344
Lamont, OK	MD4-1000	14 Aug 17	17:08:04	17:09:33	89
Lamont, OK	MD4-1000	14 Aug 17	17:39:45	17:53:40	835
Lamont, OK	MD4-1000	14 Aug 17	18:00:23	18:13:15	772
Lamont, OK	MD4-1000	14 Aug 17	18:40:24	18:52:38	734
Lamont, OK	MD4-1000	14 Aug 17	20:00:19	20:10:55	636
Lamont, OK	MD4-1000	14 Aug 17	20:40:36	20:53:32	776
Lamont, OK	MD4-1000	14 Aug 17	21:59:59	22:12:24	745
Lamont, OK	MD4-1000	14 Aug 17	22:38:49	22:53:20	871
Lamont, OK	MD4-1000	14 Aug 17	23:08:01	23:23:38	937
Lamont, OK	DJI S-1000	15 Aug 17	17:09:45	17:20:21	636
Lamont, OK	DJI S-1000	15 Aug 17	20:09:50	20:20:15	625
Lamont, OK	DJI S-1000	15 Aug 17	20:41:11	20:51:54	643
Lamont, OK	DJI S-1000	15 Aug 17	21:09:52	21:21:11	679
Lamont, OK	DJI S-1000	15 Aug 17	21:41:12	21:52:07	655
Lamont, OK	DJI S-1000	15 Aug 17	22:10:04	22:21:10	666
Lamont, OK	DJI S-1000	15 Aug 17	22:41:39	22:52:34	655
Lamont, OK	DJI S-1000	15 Aug 17	23:09:58	23:21:01	663
Lamont, OK	MD4-1000	15 Aug 17	17:13:37	17:24:14	637
Lamont, OK	MD4-1000	15 Aug 17	17:37:56	17:40:38	162
Lamont, OK	MD4-1000	15 Aug 17	20:08:27	20:22:49	862
Lamont, OK	MD4-1000	15 Aug 17	20:38:30	20:53:57	927
Lamont, OK	MD4-1000	15 Aug 17	21:08:32	21:22:05	813
Lamont, OK	MD4-1000	15 Aug 17	21:39:47	21:52:48	781
Lamont, OK	MD4-1000	15 Aug 17	22:08:10	22:21:53	823
Lamont, OK	MD4-1000	15 Aug 17	22:38:06	22:53:26	920
Lamont, OK	DJI S-1000	17 Aug 17	16:40:06	16:50:52	646
Lamont, OK	DJI S-1000	17 Aug 17	17:09:52	17:21:02	670
Lamont, OK	DJI S-1000	17 Aug 17	17:40:07	17:50:55	648
Lamont, OK	DJI S-1000	17 Aug 17	18:10:15	18:20:51	636
Lamont, OK	DJI S-1000	17 Aug 17	18:40:05	18:50:46	641
Lamont, OK	DJI S-1000	17 Aug 17	19:10:01	19:20:35	634
Lamont, OK	DJI S-1000	17 Aug 17	19:39:55	19:50:30	635
Lamont, OK	DJI S-1000	17 Aug 17	20:10:21	20:21:30	669
Lamont, OK	DJI S-1000	17 Aug 17	20:40:05	20:50:49	644
Lamont, OK	DJI S-1000	17 Aug 17	21:09:57	21:20:50	653
Lamont, OK	DJI S-1000	17 Aug 17	21:40:23	21:51:25	662
Lamont, OK	DJI S-1000	17 Aug 17	22:10:14	22:20:47	633
Lamont, OK	DJI S-1000	17 Aug 17	22:40:00	22:46:39	399
Lamont, OK	DJI S-1000	17 Aug 17	23:09:58	23:20:44	646

**Table 3:** Meteorological measurement, sampling instrument, and sampling height(s) for the variables measured at the 10 m micrometeorological tower installed at Cullman, AL during VORTEX-SE, and at the three 10-m micrometeorological towers installed during LAFE.

Variable	Instrument	Sampling Height(s) (m AGL)
Temperature, dew point temperature	Vaisala HMP110 humidity and temperature probe	2.0
Temperature	Platinum resistance thermometer in aspirated shield	2, 10.0
Pressure	RM Young 61302V	1.0
Net radiation	Hukseflux 4-component net radiometer	2.5
Soil temperature	TP01 soil temperature probe	−0.02, −0.05, −0.10, −0.20, −0.50
Soil moisture	Vegetronix	−0.05, −0.10, −0.20
Wind speed, wind direction	RM Young propeller anemometer	2.0, 10.0
Rainfall	TB3 tipping bucket rain gauge	2.5
Latent heat flux	EC155 closed path infrared gas analyzer	2.0, 10.0
CO_2_ flux	EC155 closed path infrared gas analyzer	2.0, 10.0
Sensible heat flux	CSAT3 sonic anemometer	3.0
Photosynthetically active radiation	PAR: LI-190	2.4

**Table 4:** Summary statistics on the inter-comparisons shown in the present study between the iMet-XQ sensors’ measurements of temperature (T), relative humidity (RH), and specific humidity (q) and measurements from: (1) A 10-m micrometeorological tower, (2) rawinsondes, (3) iMet-XQ sensors on the same sUAS platform, and (4) iMet-XQ sensors on different sUAS platforms. In the table below the, mean difference, standard deviation in the difference, root mean square error, R^2^, *p*-value, and number of cases are reported. When comparing the iMet-XQ measurements with the tower measurements, the means of the data ±15 min from the time of the sUAS flight were used for computing the statistics reported below. For all other comparisons, the means were computed in 10-m height bins and used to calculate the statistics reported below.

Comparison	Variable	Date, Time	Mean Difference	Standard Deviation in Difference	Root Mean Square Error	R^2^ (p)
iMet-XQ–Tower Measurement	T	28 Apr 2017, 1600-2300 UTC	0.65 °C	0.21 °C	2.55 °C	0.98 (<0.01)
iMet-XQ–Tower Measurement	RH	28 Apr 2017, 1600-2300 UTC	1.33%	1.57%	3.65%	0.95 (<0.01)
iMet-XQ–Tower Measurement	q	28 Apr 2017, 1600-2300 UTC	0.10 g kg^−1^	0.46 g kg^−1^	0.99 g kg^−1^	0.44 (0.04)
iMet-XQ–Sonde	T	28 Apr 2017, 1700 UTC	0.59 °C	0.41 °C	4.89 °C	0.16 (0.06)
iMet-XQ–Sonde	RH	28 Apr 2017, 1700 UTC	2.63%	3.08%	16.2%	0.05 (0.31)
iMet-XQ–Sonde	q	28 Apr 2017, 1700 UTC	1.20 g kg^−1^	0.74 g kg^−1^	3.20 g kg^−1^	0.03 (0.49)
iMet-XQ–Sonde	T	28 Apr 2017, 1800 UTC	0.33 °C	0.30 °C	4.00 °C	0.16 (0.05)
iMet-XQ–Sonde	RH	28 Apr 2017, 1800 UTC	1.24%	2.70%	15.5%	0.0 (0.99)
iMet-XQ–Sonde	q	28 Apr 2017, 1800 UTC	0.70 g kg^−1^	0.44 g kg^−1^	3.07 g kg^−1^	0.06 (0.25)
iMet-XQ–Sonde	T	28 Apr 2017, 2100 UTC	0.40 °C	0.08 °C	5.50 °C	0.20 (0.03)
iMet-XQ–Sonde	RH	28 Apr 2017, 2100 UTC	0.16%	1.73%	13.08%	0.03 (0.42)
iMet-XQ–Sonde	q	28 Apr 2017, 2100 UTC	0.51 g kg^−1^	0.41 g kg^−1^	2.95 g kg^−1^	0.04 (0.39)
iMet-XQ–Sonde	T	28 Apr 2017, 2300 UTC	−0.28 °C	0.13 °C	5.56 °C	0.21 (0.03)
iMet-XQ–Sonde	RH	28 Apr 2017, 2300 UTC	−0.32%	0.96%	13.02%	0.10 (0.14)
iMet-XQ–Sonde	q	28 Apr 2017, 2300 UTC	−0.10 g kg^−1^	0.18 g kg^−1^	2.96 g kg^−1^	0.14 (0.07)
iMet-XQ Sensor1–iMet-XQ Sensor2	T	5 Apr 2017, 1238 UTC	−0.13 °C	0.10 °C	0.17 °C	0.98 (<0.01)
iMet-XQ Sensor1–iMet-XQ Sensor2	RH	5 Apr 2017, 1238 UTC	3.85%	0.75%	3.92%	0.90 (<0.01)
iMet-XQ Sensor1–iMet-XQ Sensor2	q	5 Apr 2017, 1238 UTC	0.40 g kg^−1^	0.05 g kg^−1^	0.40 g kg^−1^	>0.99 (<0.01)
iMet-XQ Sensor1–iMet-XQ Sensor2	T	5 Apr 2017, 1628 UTC	0.15 °C	0.08 °C	0.17 °C	0.99 (<0.01)
iMet-XQ Sensor1–iMet-XQ Sensor2	RH	5 Apr 2017, 1628 UTC	3.75%	0.39%	3.77%	0.98 (<0.01)
iMet-XQ Sensor1–iMet-XQ Sensor2	q	5 Apr 2017, 1628 UTC	0.66 g kg^−1^	0.09 g kg^−1^	0.66 g kg^−1^	0.72 (<0.01)
iMet-XQ Sensor1–iMet-XQ Sensor2	T	5 Apr 2017, 1713 UTC	0.01 °C	0.15 °C	0.15 °C	0.97 (<0.01)
iMet-XQ Sensor1–iMet-XQ Sensor2	RH	5 Apr 2017, 1713 UTC	3.13%	0.96%	3.26%	0.95 (<0.01)
iMet-XQ Sensor1–iMet-XQ Sensor2	q	5 Apr 2017, 1713 UTC	0.49 g kg^−1^	0.16 g kg^−1^	0.52 g kg^−1^	0.67 (<0.01)
iMet-XQ Sensor1–iMet-XQ Sensor2	T	5 Apr 2017, 1823 UTC	0.14 °C	0.14 °C	0.19 °C	0.97 (<0.01)
iMet-XQ Sensor1–iMet-XQ Sensor2	RH	5 Apr 2017, 1823 UTC	1.37%	0.56%	1.47%	0.85 (<0.01)
iMet-XQ Sensor1–iMet-XQ Sensor2	q	5 Apr 2017, 1823 UTC	0.33 g kg^−1^	0.12 g kg^−1^	0.35 g kg^−1^	0.76 (<0.01)
iMet-XQ Sensor1–iMet-XQ Sensor2	T	5 Apr 2017, 1929 UTC	0.17 °C	0.13 °C	0.22 °C	0.98 (<0.01)
iMet-XQ Sensor1–iMet-XQ Sensor2	RH	5 Apr 2017, 1929 UTC	3.34%	1.08%	3.50%	0.88 (<0.01)
iMet-XQ Sensor1–iMet-XQ Sensor2	q	5 Apr 2017, 1929 UTC	0.81 g kg^−1^	0.21 g kg^−1^	0.84 g kg^−1^	0.36 (<0.01)
iMet-XQ Sensor1–iMet-XQ Sensor2	T	5 Apr 2017, 2027 UTC	0.00 °C	0.08 °C	0.08 °C	>0.99 (<0.01)
iMet-XQ Sensor1–iMet-XQ Sensor2	RH	5 Apr 2017, 2027 UTC	1.91%	0.80%	2.07%	0.81 (<0.01)
iMet-XQ Sensor1–iMet-XQ Sensor2	q	5 Apr 2017, 2027 UTC	0.40 g kg^−1^	0.16 g kg^−1^	0.43 g kg^−1^	0.45 (<0.01)
iMet-XQ DJI S-1000–iMet-XQ MD4-1000	T	28 Apr 2017, 1641 UTC	0.12 °C	0.12 °C	0.16 °C	0.97 (<0.01)
iMet-XQ DJI S-1000–iMet-XQ MD4-1000	RH	28 Apr 2017, 1641 UTC	2.81%	0.87%	2.87%	0.91 (<0.01)
iMet-XQ DJI S-1000–iMet-XQ MD4-1000	q	28 Apr 2017, 1641 UTC	0.63 g kg^−1^	0.16 g kg^−1^	0.63 g kg^−1^	0.11 (0.13)
iMet-XQ DJI S-1000–iMet-XQ MD4-1000	T	28 Apr 2017, 1706 UTC	0.24 °C	0.16 °C	0.28 °C	0.97 (<0.01)
iMet-XQ DJI S-1000–iMet-XQ MD4-1000	RH	28 Apr 2017, 1706 UTC	6.80%	2.91%	9.08%	0.44 (<0.01)
iMet-XQ DJI S-1000–iMet-XQ MD4-1000	q	28 Apr 2017, 1706 UTC	1.58 g kg^−1^	0.72 g kg^−1^	1.70 g kg^−1^	0.002 (0.84)
iMet-XQ DJI S-1000–iMet-XQ MD4-1000	T	28 Apr 2017, 1808 UTC	0.21 °C	0.09 °C	0.22 °C	0.99 (<0.01)
iMet-XQ DJI S-1000–iMet-XQ MD4-1000	RH	28 Apr 2017, 1808 UTC	7.30%	2.54%	7.54%	0.76 (<0.01)
iMet-XQ DJI S-1000–iMet-XQ MD4-1000	q	28 Apr 2017, 1808 UTC	1.69 g kg^−1^	0.52 g kg^−1^	1.73 g kg^−1^	0.15 (0.08)
iMet-XQ DJI S-1000–iMet-XQ MD4-1000	T	28 Apr 2017, 1952 UTC	0.39 °C	0.17 °C	0.42 °C	0.96 (<0.01)
iMet-XQ DJI S-1000–iMet-XQ MD4-1000	RH	28 Apr 2017, 1952 UTC	7.9%	2.3%	13.67%	0.68 (<0.01)
iMet-XQ DJI S-1000–iMet-XQ MD4-1000	q	28 Apr 2017, 1952 UTC	2.18 g kg^−1^	0.60 g kg^−1^	2.21 g kg^−1^	0.09 (0.17)
