# Correction: Lee, T. R., *et al.* On the Use of Rotary-Wing Aircraft to Sample Near-Surface Thermodynamic Fields: Results from Recent Field Campaigns. *Sensors* 2019, *19*(1), 10

**DOI:** 10.3390/s19092197

**Published:** 2019-05-13

**Authors:** Temple R. Lee, Michael Buban, Edward Dumas, C. Bruce Baker

**Affiliations:** 1 Cooperative Institute for Mesoscale Meteorological Studies, Norman, OK 73072, USA; michael.buban@noaa.gov; 2 NOAA ARL Atmospheric Turbulence and Diffusion Division, Oak Ridge, TN 37830, USA; ed.dumas@noaa.gov (E.D.); bruce.baker@noaa.gov (C.B.B.); 3 Oak Ridge Associated Universities, Oak Ridge, TN 37830, USA

The authors wish to make the following correction to this paper [1]. Replace Figure 11: 
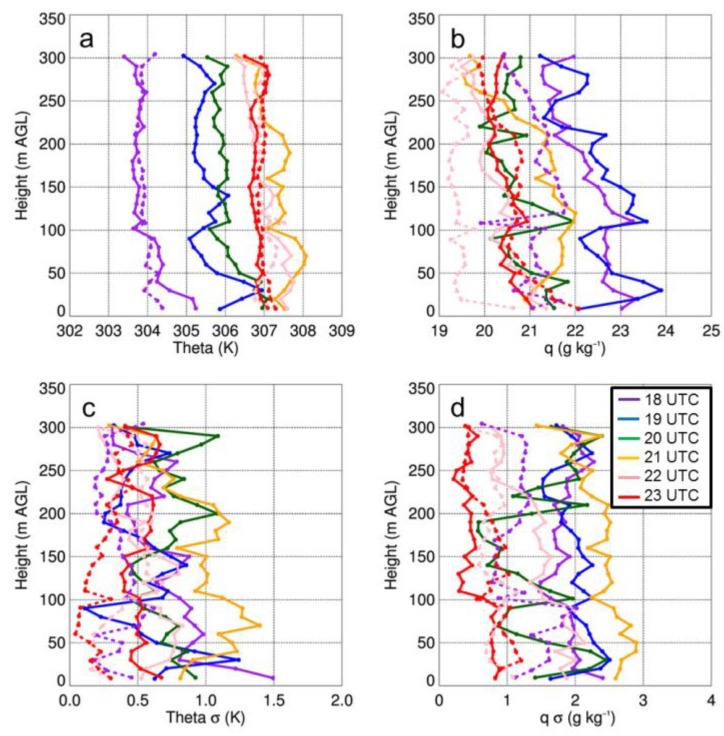
 with 
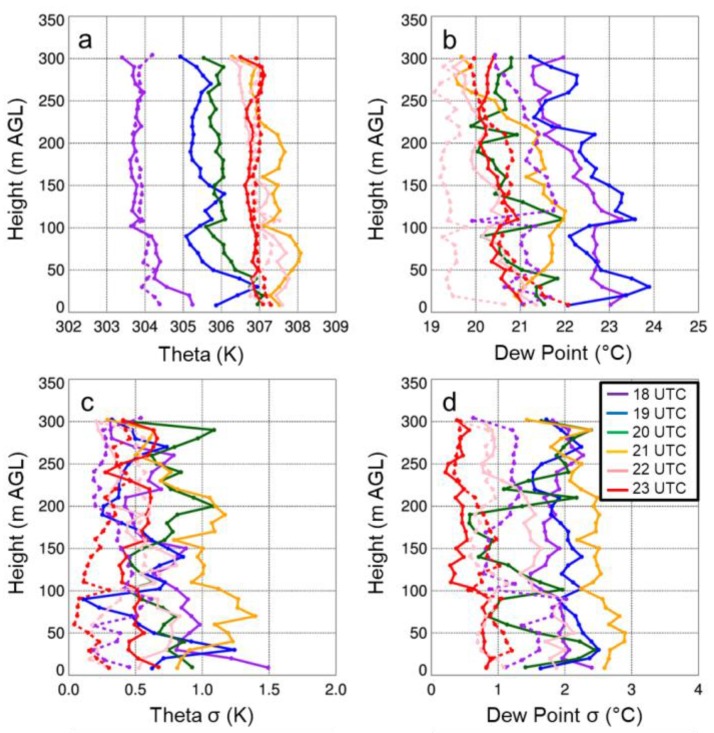


Replace Figure 12 
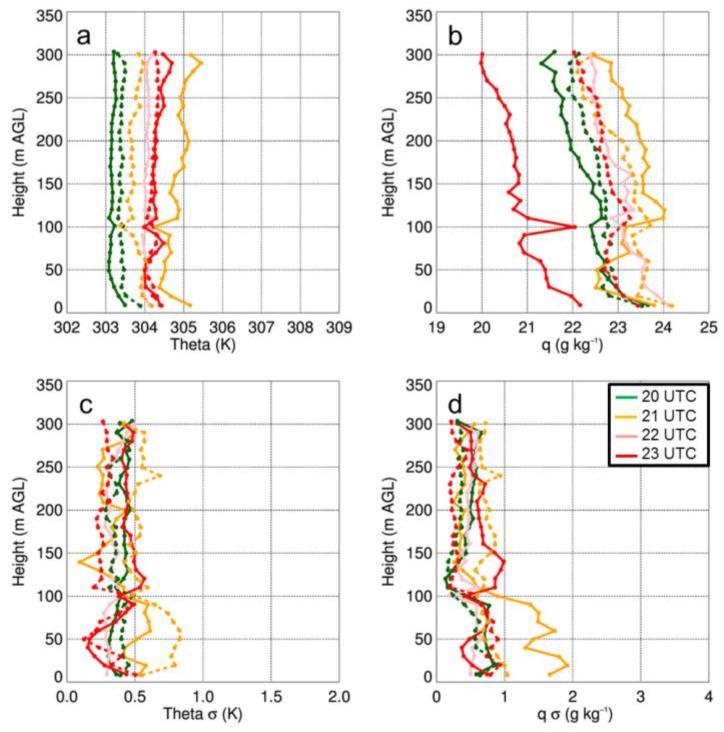
 with 
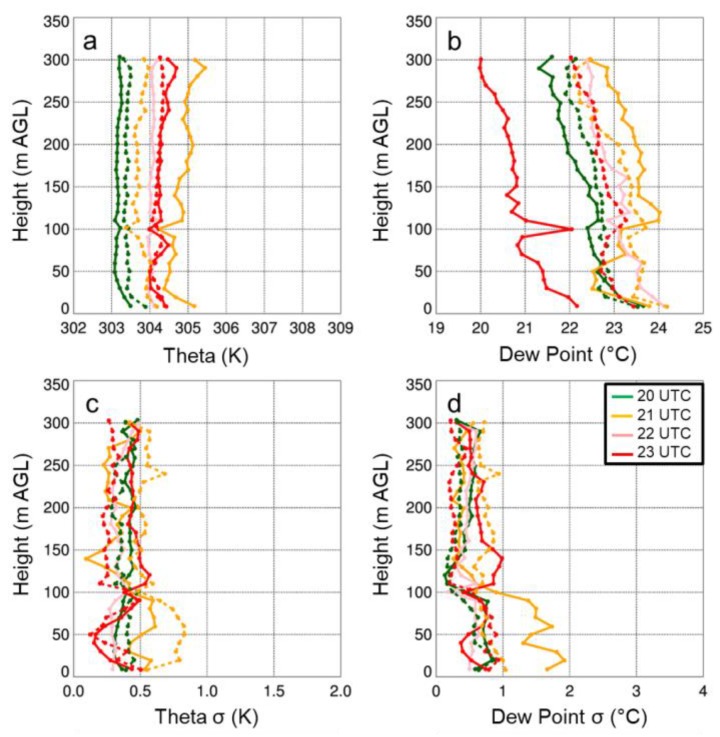


Replace Figure 13 
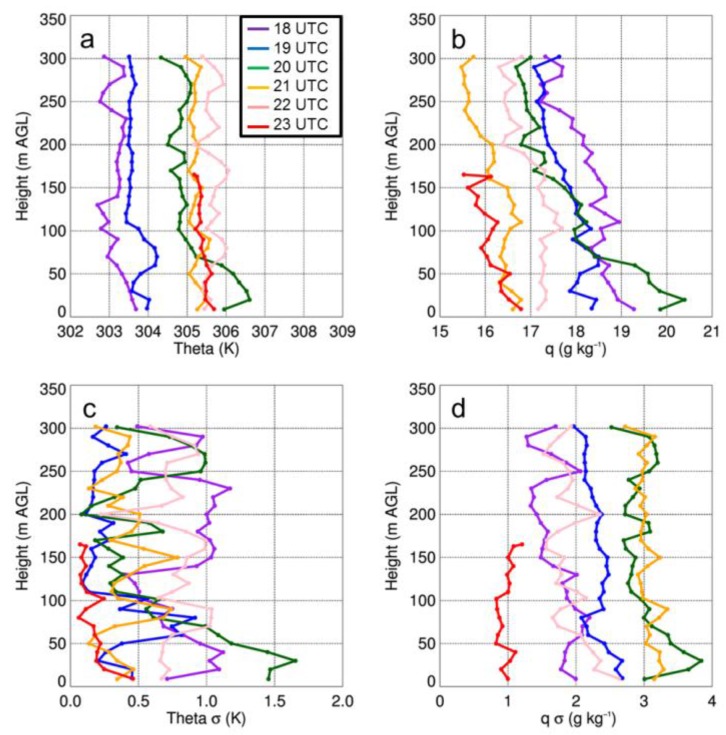
 with 
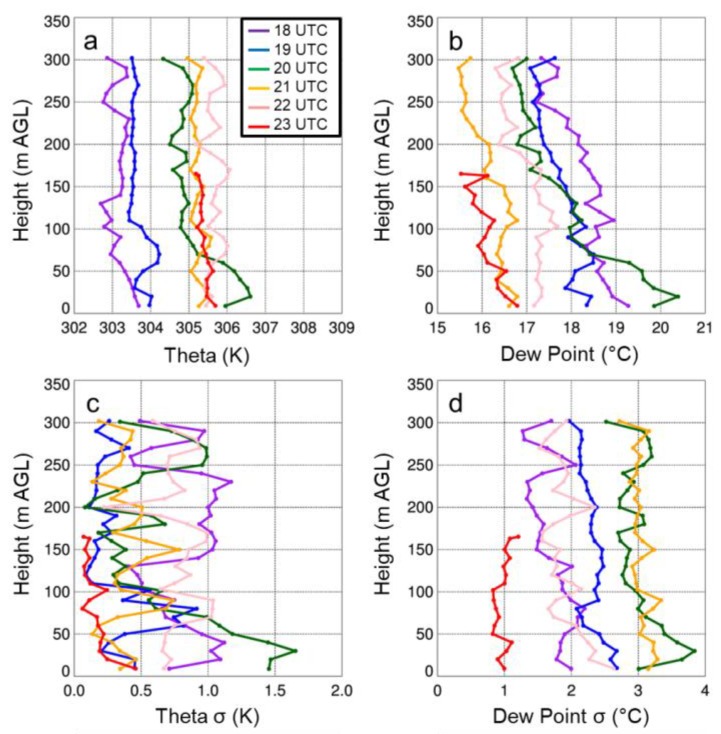


Replace Figure 14 
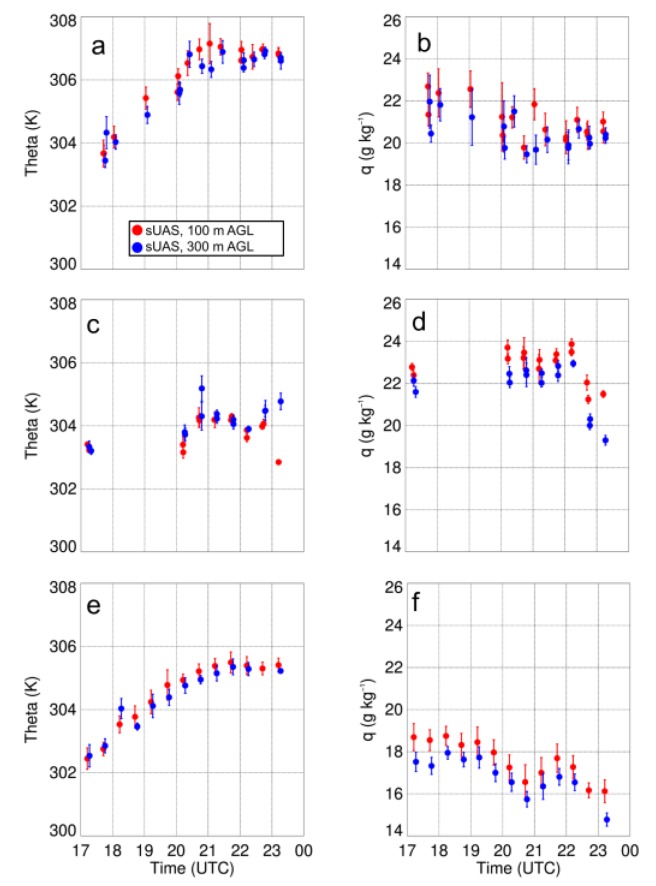
 with 
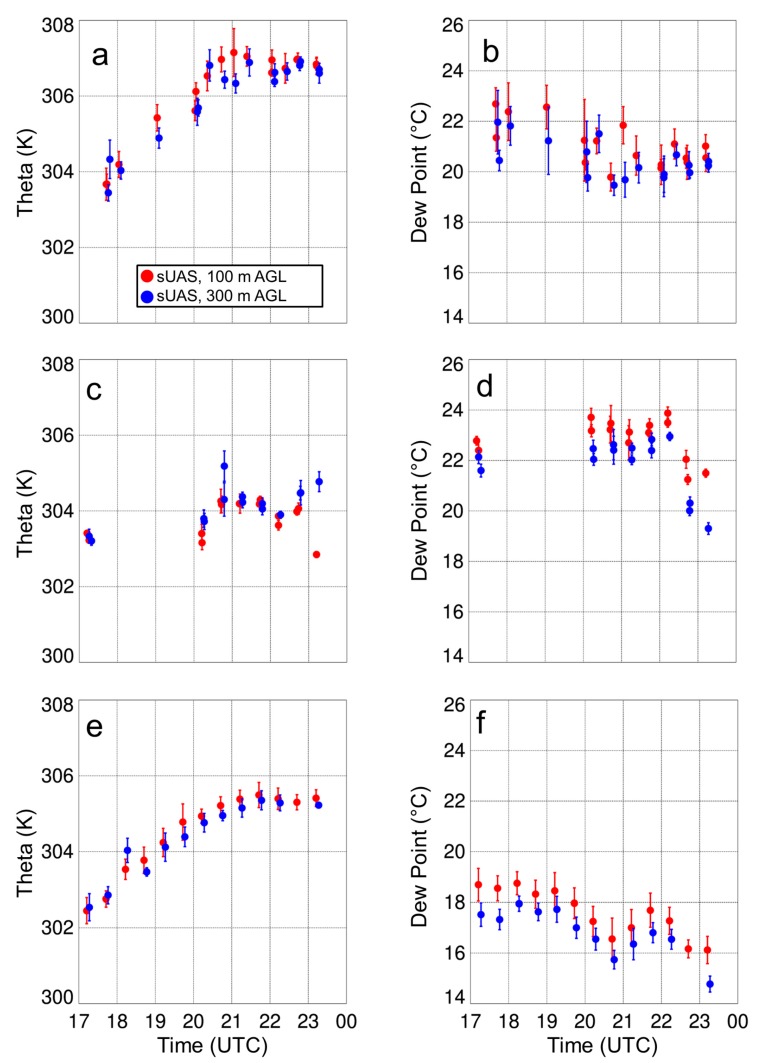


As a result of the changes to the axes, the figure caption for Figure 11 that reads “Vertical profiles of potential temperature (i.e., theta) (a) and q (b) as a function of height for select sUAS flights with the DJI S-1000 (solid) and MD4-1000 sUAS (dashed) on 14 August 2017. Panels (c) and (d) show the standard deviation (σ) in potential temperature and q, respectively…” should be replaced with:

“Vertical profiles of potential temperature (i.e., theta) (a) and dew point (b) as a function of height for select sUAS flights with the DJI S-1000 (solid) and MD4-1000 sUAS (dashed) on 14 August 2017. Panels (c) and (d) show the standard deviation (σ) in potential temperature and dew point, respectively...”

The figure caption for Figure 12 that reads “Vertical profiles of potential temperature (a) and specific humidity (b) as a function of height for select sUAS flights with the DJI S-1000 (solid) and MD4-1000 (dashed) sUAS on 15 August 2017. Panels (c) and (d) show the standard deviation (σ) in potential temperature and specific humidity, respectively. …” should be replaced with:

“Vertical profiles of potential temperature (a) and dew point (b) as a function of height for select sUAS flights with the DJI S-1000 (solid) and MD4-1000 (dashed) sUAS on 15 August 2017. Panels (c) and (d) show the standard deviation in potential temperature and dew point, respectively…”

The figure caption for Figure 13 that reads “Vertical profiles of potential temperature (a) and specific humidity (b) as a function of height for select sUAS flights with the DJI S-1000 on 17 August 2017. Panels (c) and (d) show the standard deviation (σ) in potential temperature and specific humidity, respectively…” should be replaced with:

“Vertical profiles of potential temperature (a) and dew point (b) as a function of height for select sUAS flights with the DJI S-1000 on 17 August 2017. Panels (c) and (d) show the standard deviation in potential temperature and dew point, respectively…”

The figure caption for Figure 14 that reads “Mean and standard deviation of potential temperature (a) and q…” should be replaced with:

“Mean and standard deviation of potential temperature (a) and dew point (b)…”

The first sentence on page 18, i.e., “Vertical profiles of temperature and moisture indicated a well-mixed ABL that occurred during the afternoon on all three days (Figures 11a,12a,13a), as evident by vertical gradients in potential temperature and specific humidity, which were generally <1 K and <2 g kg^−1^.” should be replaced with:

“Vertical profiles of temperature and moisture indicated a well-mixed ABL that occurred during the afternoon on all three days (Figures 11,12,13), as evident by vertical gradients in potential temperature and dew point temperature (Tdew) which were generally <1 K and <2 °C.”

The final sentence of that same paragraph, “Water vapor showed larger variability than temperature, with differences between measurements from the DJI S-1000 and MD4-1000 of up to 2 g kg^−1^.” should be replaced with:

“Dew point temperature showed larger variability than temperature, with differences between measurements from the DJI S-1000 and MD4-1000 of up to 2 °C.”

The fifth sentence of the paragraph on page 19 “Furthermore, we did not find large differences in the iMet-XQ measurements 100-m or 300-m AGL when the sUAS was flying over surfaces with different thermal characteristics (shown in Figure 9); the temperature and moisture variations remained within ±0.5 K and 0.5 g kg^−1^, respectively, during these horizontal transects.” should be replaced with:

“Furthermore, we did not find large differences in the iMet-XQ measurements 100-m or 300-m AGL when the sUAS was flying over surfaces with different thermal characteristics (Figure 9); the potential temperature and dew point variations remained within ±0.5 K and 0.5 °C, respectively, during these horizontal transects.”

The conclusions of the study are unchanged.
